# Trends in Pregnancy-Associated Opioid Toxicity and Mortality

**DOI:** 10.23889/ijpds.v9i5.2810

**Published:** 2024-09-18

**Authors:** David Clark

**Affiliations:** 1 Public Health Scotland

## Objectives

To examine trends in pregnancy-associated non-fatal and fatal opioid toxicity and all-cause mortality, and identify associated factors.

## Approach

We conducted a population-based study of 1,555,370 pregnancies in Ontario, Canada, 2013-2022. We analyzed linked administrative datasets, including coroner data, and calculated pregnancy-associated (during pregnancy or within one-year post-pregnancy) non-fatal/fatal opioid toxicity and all-cause mortality ratios per 100,000 livebirths by year and timing (pregnancy, post-pregnancy). Poisson regression models analyzed trends in outcomes and generated adjusted relative risks (aRR) of opioid toxicity by socio-demographic and clinical factors.

## Results

Pregnancy-associated non-fatal opioid toxicity increased 220% between 2013 and 2020 (45.5-145.4/100,000 livebirths) before declining by 30% in 2021. Over the study period, fatal opioid toxicity increased 150% (6.8-17.5/100,000) and all-cause mortality increased 120% (32.8-71.2/100,000). Our methods did not identify any opioid toxicity deaths in pregnancy, and most non-fatal (66.6%) and fatal (88.9%) opioid toxicity and all-cause mortality (73.9%) occurred 43-365 days post-pregnancy. The percent of deaths attributed to opioids increased from 12.7% in 2015 to 25.0% in 2020. Substance use disorder (aRR 19.52, 95% CI 16.87-22.58), pre-pregnancy opioid toxicity (aRR 4.69, 3.81-5.78), mental illness (aRR 2.01, 1.75-2.29), high neighbourhood deprivation (aRR 1.45, 1.28-1.64), and social disadvantage (aRR 3.21, 2.77-3.71) were associated with elevated risk of opioid toxicity.

## Conclusions

Pregnancy-associated opioid toxicity and mortality have increased substantially. In 2020, 1 in 4 pregnancy-associated deaths involved opioids.

## Implications

Findings highlight the need for comprehensive care and perinatal harm reduction services. System-level improvements to reduce poor outcomes must include complete data capture of all pregnancy-associated deaths.

